# Comparison of low-dose CT and MRI in enthesitis-related arthritis patients with sacroiliitis

**DOI:** 10.1093/bjr/tqae210

**Published:** 2024-10-17

**Authors:** Yunus Emre Bayrak, Törehan Özer, Yonca Anık, Nihal Şahin, Hafize Emine Sönmez

**Affiliations:** Department of Pediatric Rheumatology, Kocaeli University, Kocaeli, 41100, Turkey; Department Radiology, Kocaeli University, Kocaeli, 41100, Turkey; Department Radiology, Kocaeli University, Kocaeli, 41100, Turkey; Department of Pediatric Rheumatology, Kocaeli University, Kocaeli, 41100, Turkey; Department of Pediatric Rheumatology, Kocaeli University, Kocaeli, 41100, Turkey

**Keywords:** sacroiliitis, low-dose CT, bone marrow oedema, erosion

## Abstract

**Objective:**

This study investigated the utility of low-dose CT (ldCT) compared with MRI in diagnosing sacroiliitis in enthesitis-related arthritis (ERA) patients.

**Methods:**

Thirty patients diagnosed with ERA were evaluated, with a median follow-up of 1.47 years. Images from patients were examined by two paediatric radiologists. For each patient, we assessed the density changes on ldCT at corresponding locations, employing the signal intensity observed on MRI across each joint surface as a reference. While measurements in areas without oedema on MRI showed relatively high density, measurements in areas with oedema on MRI showed relatively low density.

**Results:**

MRI revealed bilateral bone marrow oedema in 22 (73.3%) patients. During the ldCT evaluation of the right iliac crest, lower density was identified on ldCT in regions displaying heightened signal intensity on MRI in 20 (66.6%) patients. On the right sacral side, lower density was observed in the ldCT of 22 (73.3%) patients. Moving to the left iliac crest, 18 (60%) patients displayed a lower density. On the left sacral side, lower density was identified on ldCT in 22 (73.3%) patients. Erosion was detected in 23 patients on ldCT, whereas only 11 patients showed erosion on MRI.

**Conclusions:**

This study suggests that ldCT is superior to MRI for early structural change detection. Pixel-based density evaluation in ldCT aligns with MRI for bone marrow oedema.

**Advances in knowledge:**

The present study showed that ldCT is superior to MRI for early structural change detection. Pixel-based density evaluation in ldCT aligns with MRI findings for bone marrow oedema.

## Introduction

Juvenile idiopathic arthritis (JIA) is the predominant cause of chronic arthritis in childhood, encompassing a diverse spectrum of diagnoses with seven distinct subgroups.^1^ Among these, enthesitis-related arthritis (ERA) constitutes 18.9% of JIA cases in our region, predominantly affecting boys over the age of 6.^2^ ERA mainly targets the joints and entheses of the lower extremities, potentially progressing to the spine or sacroiliac (SI) joints. It is characterized by the absence of rheumatoid factor (RF) and is strongly associated with human leukocyte antigen-B27 (HLA-B27).^2^ SI involvement is a common manifestation of ERA, with patients typically presenting with lower back pain that worsens during periods of inactivity. Unfortunately, conventional plain radiographs are inadequate for diagnosing sacroiliitis in childhood. Although MRI is the most sensitive radiological method for detecting sacroiliitis, it comes with challenges such as prolonged procedure times and the need for sedation in some paediatric cases.^3^ Recent findings from adult studies suggest that low-dose CT (ldCT) may offer a viable alternative for diagnosing sacroiliitis.^3^^,^^4^ ldCT examinations, administered at radiation rates comparable to those used in radiography, have been proven to be superior to radiography in detecting structural lesions. ldCT of the sacroiliac joints consistently results in an effective dose of less than 1 mSv. This level of radiation places it in the minimal risk category (0.1-1 mSv), comparable to sacroiliac joint radiography. CT scans can be more precisely collimated than radiographs, potentially leading to lower gonadal radiation exposure with ldCT. In pelvic or sacroiliac joint radiography, women's gonads are directly exposed to the primary beam, and men's gonads are typically within it during pelvic radiographs. While sacroiliac joint projections usually avoid this, the male gonads remain close to the beam in the AP Ferguson view. In contrast, ldCT allows better control of the exposed area, keeping male gonads further from the beam and sometimes excluding female gonads entirely. This is particularly important for young adults of reproductive age. Women should also empty their bladder before ldCT to further reduce exposure if the ovaries are low in the pelvis. However, there is no data on the usefulness of ldCT for detecting sacroiliitis during childhood. Therefore, the aim of the stud to evaluate the effectiveness of ldCT in detecting sacroiliitis in paediatric cases compared with MRI.

## Methods

Patients who were diagnosed with ERA and accompanied by sacroiliitis were enrolled in the study. The diagnosis of ERA is established using the classification criteria set forth by the International League of Associations for Rheumatology (ILAR).^1^

Demographic data, clinical manifestations, and laboratory findings, including white blood cell (WBC) count, erythrocyte sedimentation rate (ESR), C-reactive protein (CRP), and HLA-B27 were meticulously documented, alongside a comprehensive examination of treatment modalities. The study also investigated the following parameters in these patients: (1) inflammatory back pain lasting more than three months, (2) morning stiffness, (3) enthesitis, (4) Schober’s test, (5) Patrick-Faber test, and tenderness on pressure of the pelvis to evaluate the presence of sacroiliitis, (6) uveitis, and (7) familial history of HLA-B27-associated disease. The exclusion criteria were patients with psoriasis, familial Mediterranean fever, and inflammatory bowel disease.

Disease activity was assessed using the Juvenile Spondyloarthritis Disease Activity Index (JSpADA).^5^

The study adhered to the Helsinki Declaration guidelines for medical research involving humans and received approval from the local Ethics Committee. Informed consent was obtained from each patient and/or their parents.

### ldCT protocol

To minimize temporal variations, the ldCT and MRI were conducted within the same week. The CT scan was performed using a 640-MSCT device, with patients being exposed to an average effective dose of 0.615 mSv (Canon One Aquilion Genesis Edition, dynamic volume CT, Canon Medical systems Otowara Japan, 640 layer, detector 0.5 320) (voltage 120 kV, electricity was determined automatically according to patient’s volume as 80-95 mAs, rotation time 0.5 s, layer thickness 0.5 mm, reconstruction interval 2.5 mm, window width 2700 Hounsfield units [HU], and window level 350 HU). Patients did not receive any intravenous contrast agent. Compared to the radiographs at our centre, the parameters for the sacroiliac joint radiograph and the effective dose the patient is exposed to 120 kV, 80 mA, 500 ms, with FOV arranged for each patient. The average dose per scan was 0.46 mSv.

Density changes were evaluated by using the HU. The HU serves as a relative quantitative measure of radio density and is employed by radiologists in the analysis of CT images. During CT reconstruction, the absorption/attenuation coefficient of radiation within the tissue is used to generate a greyscale image. The physical density of the tissue corresponds to the absorption/attenuation of the X-ray beam. The HU is calculated through a linear transformation of the baseline linear attenuation coefficient of the X-ray beam. Distilled water (at standard temperature and pressure) is arbitrarily set at zero HU, while air is defined as −1000 HU. Upper limits can extend to 1000 for bones, 2000 for dense structures such as cochlea, and exceed 3000 for metals such as steel or silver. This linear transformation results in a Hounsfield scale displayed in grey. Denser tissue, absorbing more X-ray beams, exhibits positive values and appears bright, whereas less dense tissue, with lower X-ray absorption, displays negative values and appears dark.^6^ Measurements were conducted within regions of subchondral oedema identified on MRI. A circular region of interest (ROI) with an area of 10 mm^2^ was delineated on CT scans. Care was taken to exclude areas that exhibited a sclerotic appearance during the measurement process. This precautionary step aimed to ensure the accuracy of the density assessment because measuring from sclerotic regions could obscure the anticipated decrease in density attributed to the presence of oedema.

### MRI procedure

All MRI was examined using a 1.5 T scanner (GE Signa voyager premier edition 1.5 T, GE Healthcare, United States) with either a torso or abdomen coil. The MRI protocol comprised a fat-suppressed axial T2-weighted sequence (TR/TE 2500/85 msec, echo train length 16), coronal and axial T1-weighted sequence (TR/TE 610/minimum msec, echo train length 3), coronal STIR sequence (TR/TE 4250/42 msec, echo train length 16), and axial T2-weighted sequence (TR/TE 4650/85 msec, echo train length 16). The imaging parameters were as follows: a 4-mm section thickness, a 1-mm intersection gap, a 384 × 288 matrix, and a 26 × 26 cm field of view.

Active sacroiliitis was determined by MRI according to the Assessment in Spondyloarthritis International Society (ASAS) criteria.^7^

### Comparison of ldCT and MRI

All images were evaluated by the consensus of two radiologists who were both blinded to clinical information. The presence of oedema was evaluated using coronal STIR, axial fat-suppressed T2A, and axial T1A scans. Areas that were hyperintense on STIR and fat-suppressed T2A and hypointense on T1A were defined as oedema areas. The signal degree of the areas measured on MRI were adapted on CT images where bone marrow oedema was identified on MRI. On MRI, bone marrow oedema was classified as T2A hyperintense areas. The corresponding T2A hyperintense oedema areas were identified according to the sacral interforaminal bone marrow signal as a reference for the normal bone marrow signal.^8^ Subsequently, we measured the density changes on ldCT at the corresponding locations of the areas of bone marrow oedema on MRI, employing the signal intensity observed on MRI across each joint surface as a reference. The correlation between the absence and presence of bone marrow oedema on MRI and density change on ldCT for the same anatomical areas was assessed.

Erosion was classified based on its size. If the erosion is smaller than 4 mm and present in fewer than five areas, it is defined as millimetric erosion. If the erosion is smaller than 4 mm but affects more than five areas, it is classified as diffuse millimetric erosion. Extensive erosion is defined when the erosions are larger than 4 mm in size. Fat metaplasia refers to the replacement or infiltration of subchondral bone and bone marrow with tissue exhibiting a fat signal on MRI. These areas appear hyperintense on T1- and T2-weighted images but show signal loss on T2-weighted images with fat suppression.

Joint space narrowing on radiography is generally attributed to the loss of articular cartilage. The cut-off for joint space is accepted as 3 mm in adults, but the current literature on this subject seems insufficient for the paediatric age group. As a result, we could not make a completely objective assessment. Utilizing our hospital's extensive archive, we compared normal sacroiliac joint spaces from abdominal and pelvic CT scans taken for trauma, malignancy follow-up, and other reasons with each patient’s respective age group, accepting these as normative values. Two radiologists made the final decision together.

### Statistical analysis

Statistical analyses were performed by using PSS Statistics for Windows (version 21.0; IBM, Armonk, NY, United States). Continuous variables are reported as median and interquartile range (IQR) standard deviations. A *P*-value of less than .05 was considered indicative of a significant difference.

## Results

Thirty patients diagnosed with ERA were enrolled in the study. Of these, 11 (36.7%) were girls and 19 (63.3%) were boys. Among the 30 patients, 22 (73.3%) had a family history of rheumatic disease, and the rate of consanguineous marriage was 6.7%. The median (IQR) current age and age at diagnosis were 14.44 (6.08) and 10.30 (5.85) years, respectively. The median (IQR) follow-up time was 1.47 (2.05) years.

The clinical and laboratory features of patients at the time of diagnosis are shown in Table 1. The median JSpADA was 4 (1.6) at diagnosis. All patients initially received non-steroidal anti-inflammatory drugs (NSAIDs), 27 (90%) also used disease-modifying antirheumatic drugs (DMARDs) (methotrexate = 21, sulfasalazine = 6) and 16 (53.3%) were treated with biological agents (etanercept = 14, adalimumab = 2).

**Table 1.** Clinical and laboratory features of patients at the time of diagnosis.

Lower back pain, *n* (%)	27 (90)
Hip pain, *n* (%)	25 (83.3)
Morning stiffness, *n* (%)	24 (80)
Peripheric arthritis, *n* (%)	21 (70)
Heel pain, *n* (%)	12 (40)
Enthesitis, *n* (%)	11 (36.7)
Back pain, *n* (%)	9 (30)
Uveitis, *n* (%)	0 (0)
White blood cell, median (IQR)	7410 (4270) mm³
Haemoglobin, median (IQR)	11.9 (1.8) mg/dL
Platelet count, median (IQR)	336 000 (105 000) mm³
C-reactive protein, median (IQR)	5 (18.7) mg/dL
Erythrocyte sedimentation rate, median (IQR)	21 (33) mm/h
HLA-B27 (tested in 28), *n* (%)	24 (85.7)

During radiologic evaluation, the median (IQR) counts of active joint and enthesitis were 1 (2) and 0 (0), respectively. Eighteen patients (60%) exhibited clinical sacroiliitis, and 10 (33.3%) experienced morning stiffness. The median JSpADA was 2.5 (1.3). The median CRP level was 1.3 (4.1) mg/dL, and ESR was 13 (15) mm/h. At the time of evaluation, 14 patients were on DMARDs for a median duration of 5 months, while 16 patients were receiving biological agents for a median duration of 12 months.

**Figure 1.**
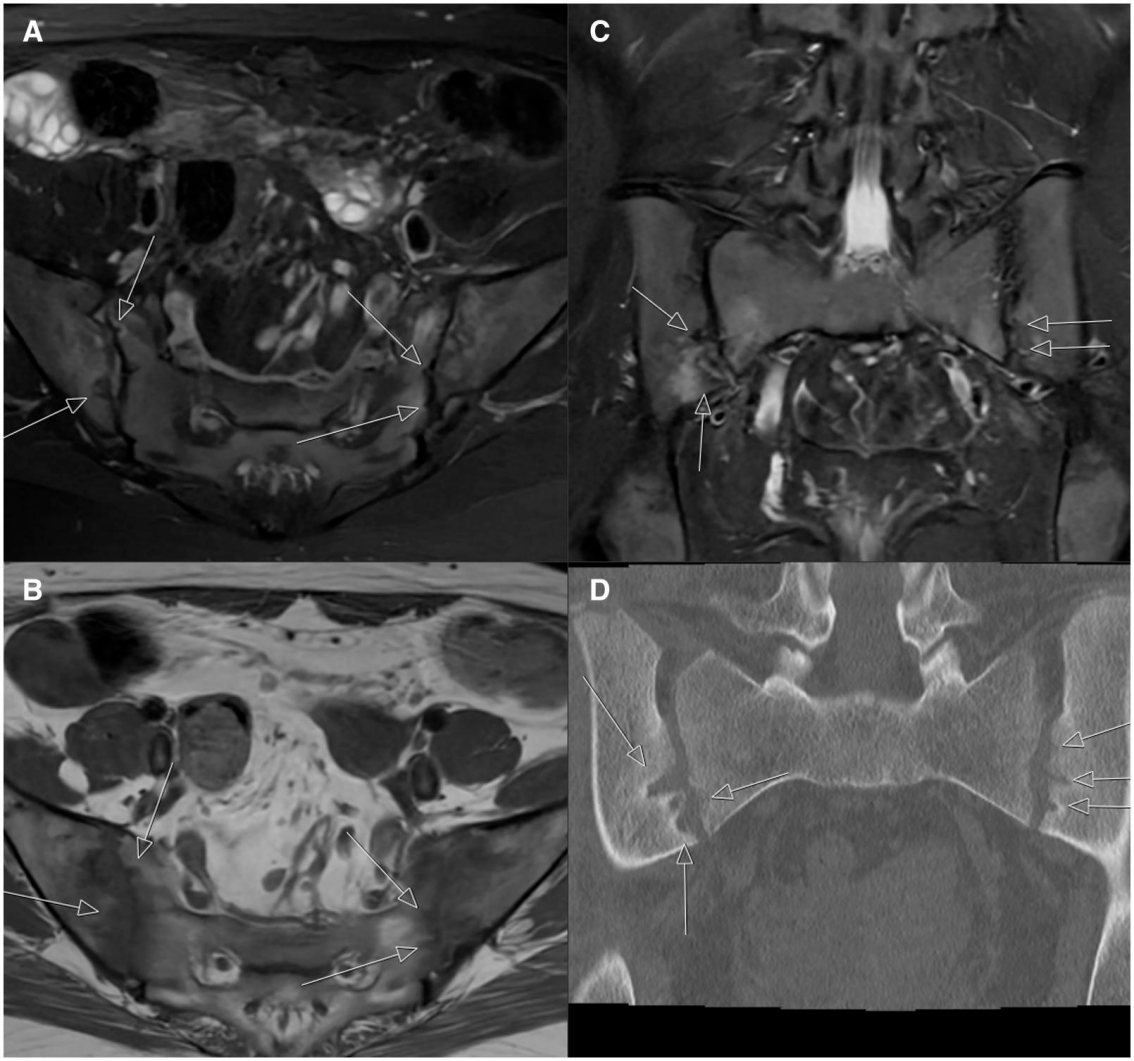
Images of widespread subchondral signal increase compatible with oedema are observed on axial fat-suppressed T2-weighted sacroiliac MR images (A), and signal loss compatible with oedema is observed in the same locations on axial non-contrast T1-weighted sacroiliac MR images (B). In the coronal STIR sequence (C), an increased subchondral signal compatible with oedema was observed. (D) Coronal image of ldCT. White arrows indicate areas of accompanying millimetric erosion. Figure emphasizes that while the erosion is noticeable on MRI, it is more clearly visible on CT.

**Figure 2.**
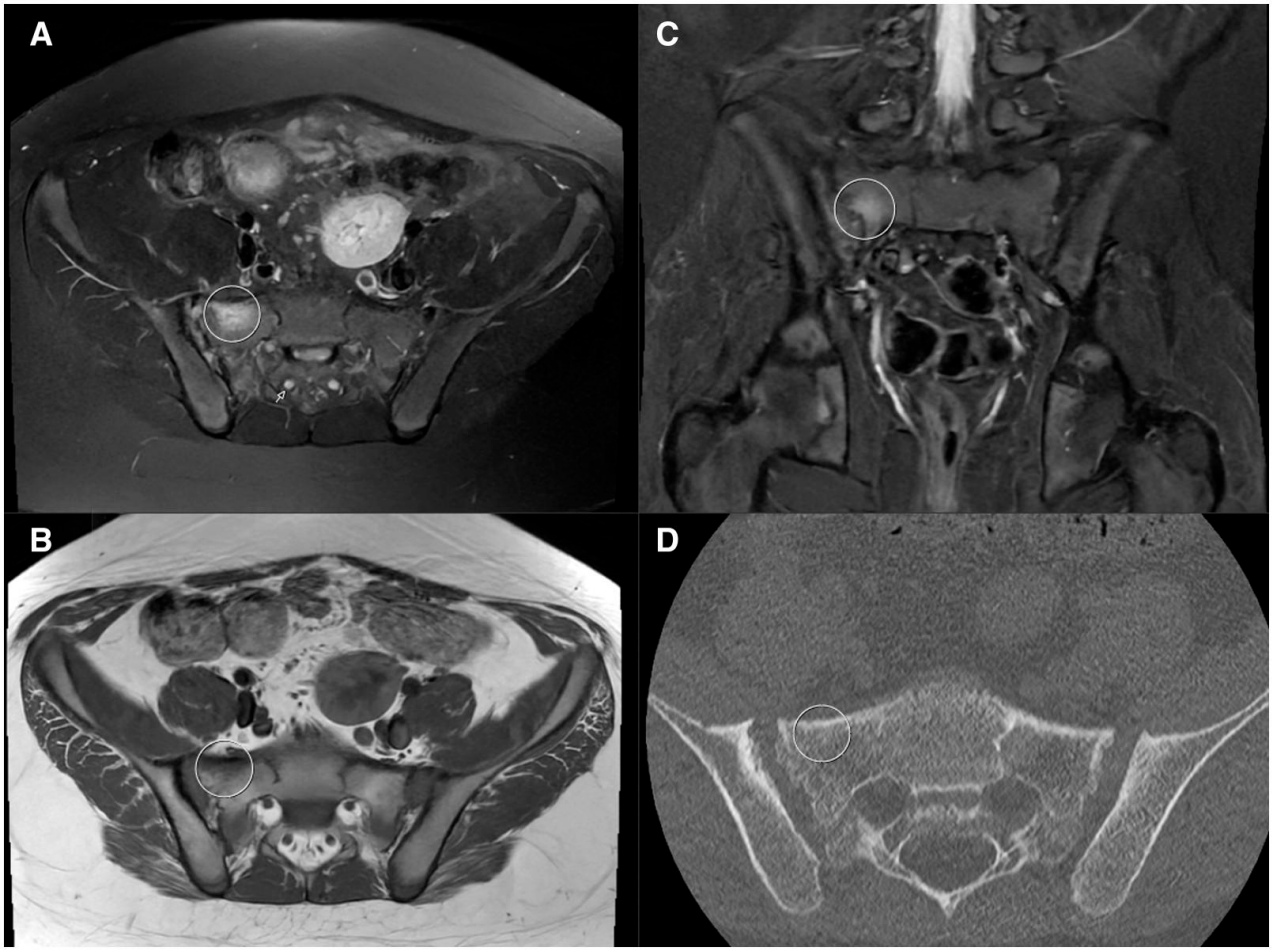
Compatible with the intense oedema area on the right sacral face, an increase in signal (circle) is observed in the axial T2-weighted fat-suppressed MRI section (A), a decrease in signal (circle) in the axial T1-weighted non-contrast MRI section (B), and an increase in signal (circle) in the coronal STIR section (C). Note that in both the T2 fat-suppressed and STIR MRI scans, the signal intensity is similar to the cerebrospinal fluid (CSF) signal in the sacral neural foramen (white arrow). (D) Axial image of ldCT.

**Figure 3.**
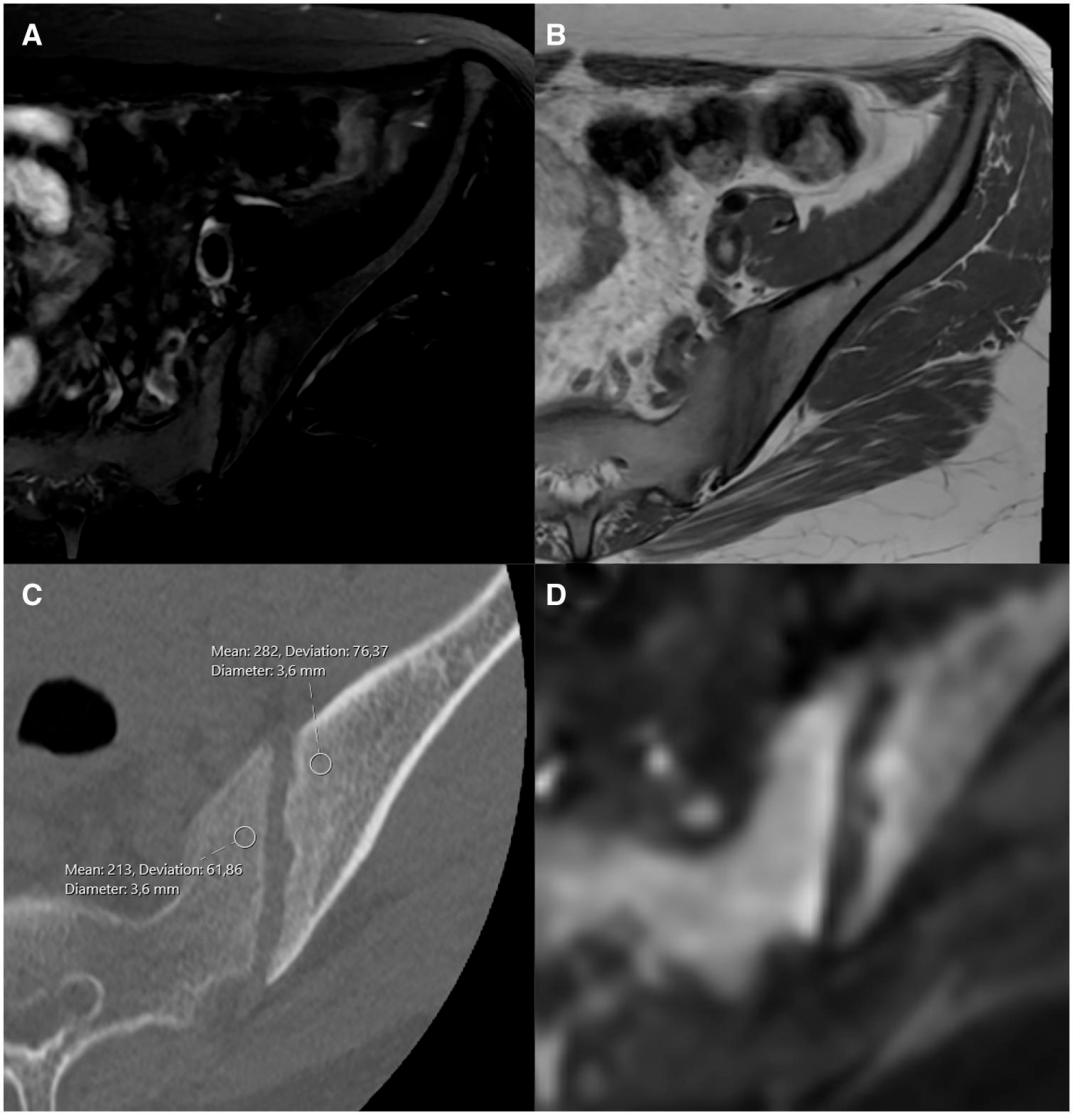
(A) Hyperintense signal on the fat-suppressed axial T2 MRI at the iliac and sacral facets of the left sacroiliac joint. (B) Hypointense signal on the axial T1 MRI at the iliac and sacral facets of the left sacroiliac joint. (C) On low-dose CT density measurements made through the ROI circle are taken in the subchondral areas of the sacral and iliac surfaces of the left sacroiliac joint. (D) Axial diffusion MRI shows signal enhancement in the subchondral area.

MRI revealed bilateral bone marrow oedema in 22 (73.3%) patients. Among them, 14 (46.7%) patients exhibited bilateral minimal bone marrow oedema, whereas seven presented with bilateral diffuse bone marrow oedema (Figures 1 and 2). The remaining patient had minimal bone marrow oedema on the right side but diffused on the left side. Bone marrow oedema was located subchondral in all patients. We assessed the density changes on ldCT at the corresponding locations of the areas of bone marrow oedema on MRI. The density of regions exhibiting bone marrow oedema (heightened signal intensity) on MRI was lower on CT, whereas the density of regions devoid of signal (no bone marrow oedema) on MRI was higher on CT. On ldCT evaluation of the right iliac crest, lower density was found on ldCT in regions exhibiting heightened signal intensity in MRI for 20 (66.6%) patients. On the right sacral side, lower density was observed on ldCT in regions exhibiting heightened signal intensity in MRI for 22 (73.3%) patients. On the left iliac crest, lower density was observed in 18 (60%) patients. On the left sacral side, lower density was observed on ldCT in regions exhibiting heightened signal intensity in MRI for 22 (73.3%) patients (Figure 3). A correlation was found between the density measurement in ldCT and the signal intensity in MRI.

**Figure 4.**
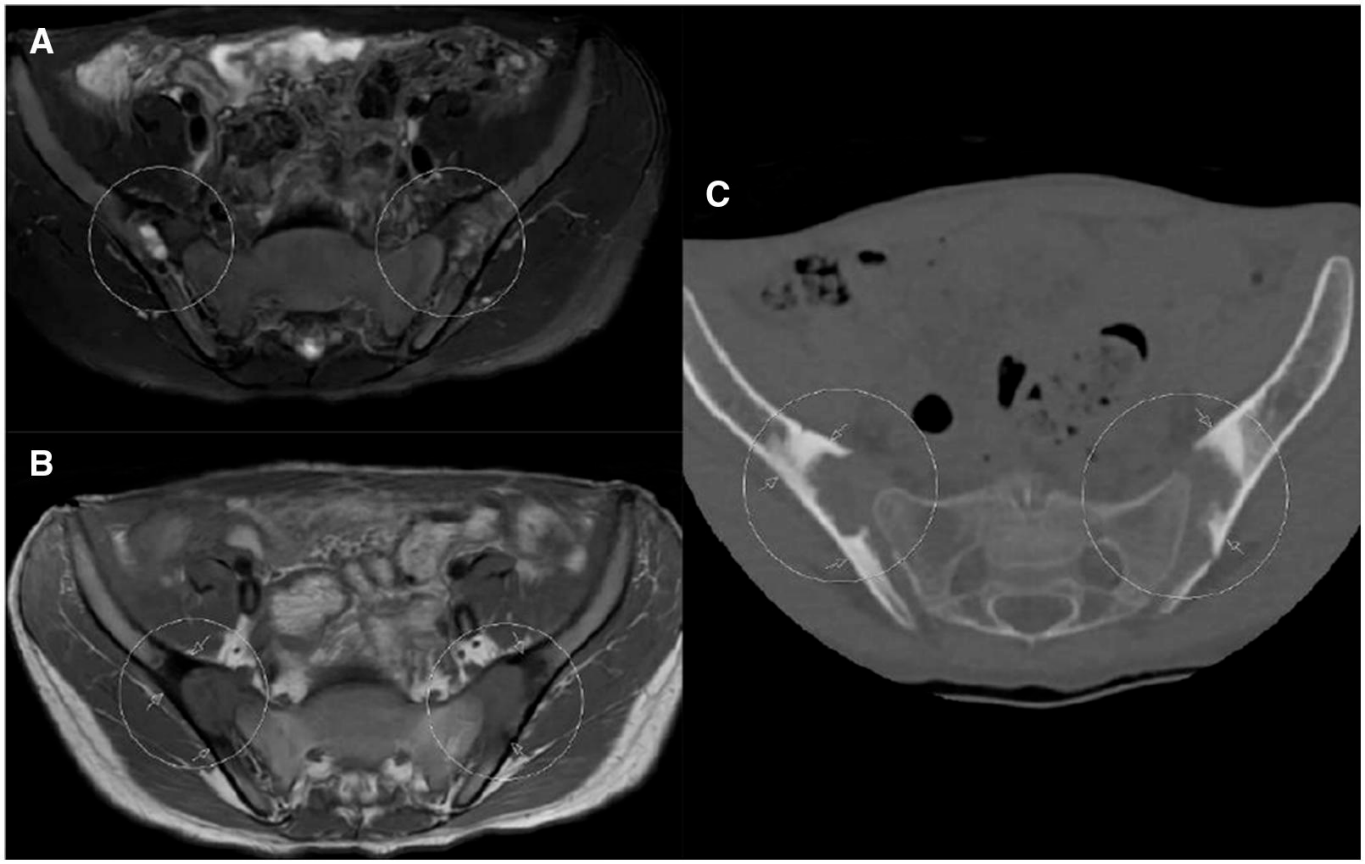
Image erosion on both MRI and ldCT. (A), T1-weighted axial MRI section (B), and ldCT section passing through the same level (white circles). Note the pseudo-widening in the joint space, which is visible in all images. In addition, observe the accompanying areas of widespread and intense sclerosis (white arrows). It is important to note that due to advanced degeneration, heterogeneous signal changes in MRI sections can occur, leading to difficulties in identifying even obvious areas of erosion. In contrast to MRI images, there is a significant contrast between erosion areas, joint space, and sclerotic areas on ldCT images.

**Figure 5.**
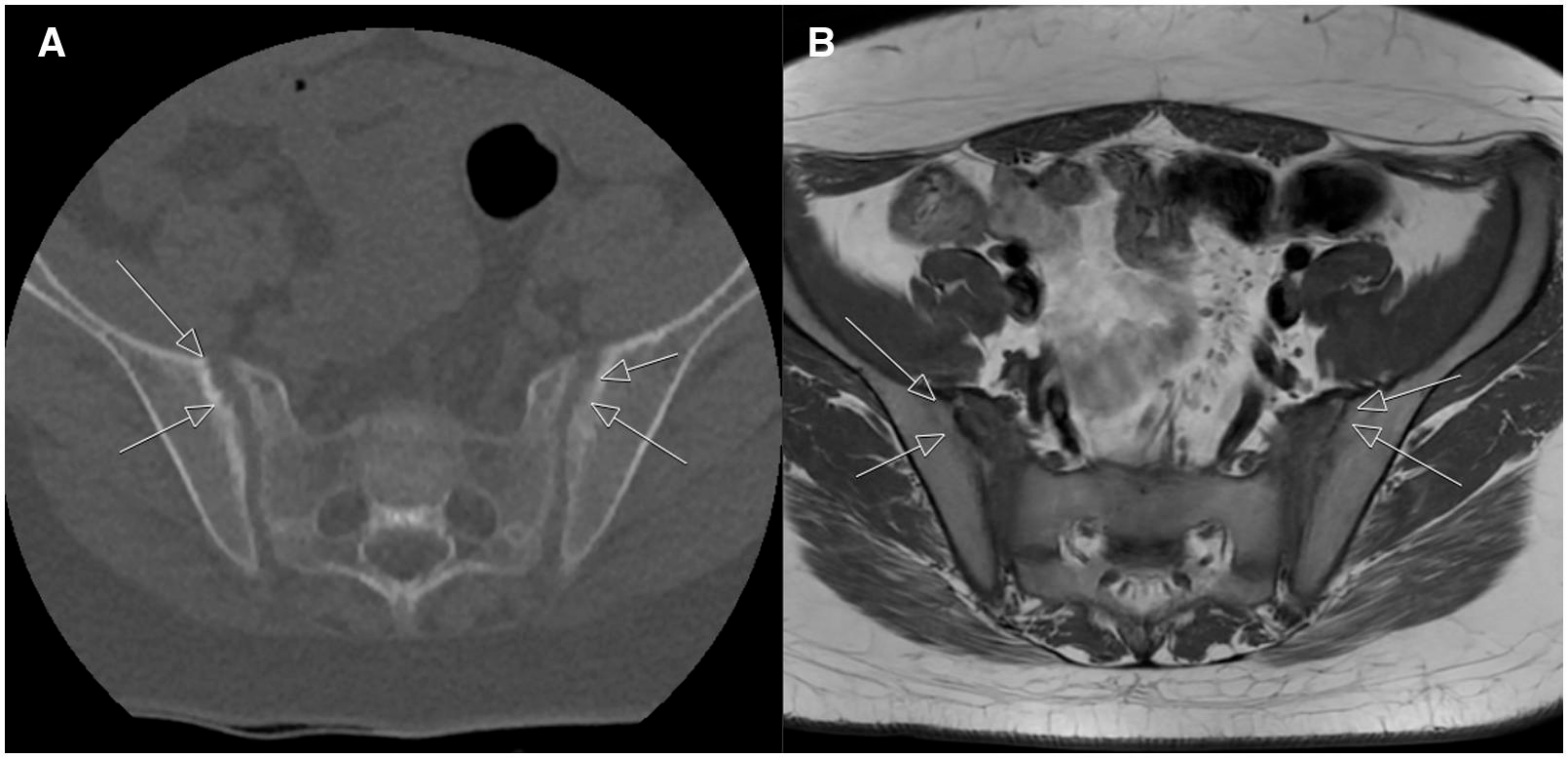
Widespread millimetric erosion is evident on CT, whereas it appears as millimetric erosion on MRI. In the axial section (A) of the low-dose CT scan of the sacroiliac joint, numerous millimetric-sized erosion areas, accompanied by sclerosis, are observed on both iliac surfaces (indicated by white arrows). In the axial non-contrast T1-weighted MRI section (B), barely visible millimetric erosions (white arrows) are noted on bilateral iliac face. When comparing sacroiliac joint images obtained from the same patient and at similar levels, erosion is much more clearly visualized on CT. In addition, many of the erosion visible on CT cannot be observed on MRI. Figures emphasizes that while the erosion is noticeable on MRI, it is clearly visible on CT.

Fat metaplasia was detected on MRI in six (20%) patients. Erosion was detected in 23 (76.6%) patients on ldCT. Erosion was minimal in nine (30%) patients, diffuse millimetric in nine (30%), and extensive in five (16.7%) patients. Erosion was detected in only 11 (36.7%) patients on MRI. Erosion was minimal in six (20%) patients, diffuse millimetric in one (3.3%), and extensive in three (13.3%) patients (Figures 4 and 5). When joint spaces are examined, on the right, it was narrowed in five patients, and on the left, it narrowed in four patients.

## Discussion

In this study, the sacroiliac MRI and ldCT results of 30 ERA patients were compared. While the signal intensity of oedema on MRI correlated with the density changes on ldCT. ldCT demonstrated superior efficacy in revealing structural lesions. To the best of our knowledge, this is the first study to compare sacroiliac ldCT and MRI in paediatric patients.

It is well known that radiographs have limited diagnostic utility in screening for sacroiliitis in children.^9^ While MRI is currently considered the gold standard for assessing the SI joint, it comes with several limitations, including extended procedure times, the requirement for sedation in certain paediatric cases, and high costs. Recent studies in adults have highlighted that ldCT may be an alternative method for assessing the SI joint. For instance, Ye et al^4^ compared the diagnostic performance of conventional radiography, ldCT, and MRI for sacroiliitis in suspected axial spondyloarthritis (axSpA) adults. They demonstrated that ldCT exhibits similar sensitivity and higher specificity compared with MRI findings for structural lesions in adults with axSpA. Additionally, the contribution of oedema on MRI and structural lesions on CT to the diagnosis in patients with non-radiographic axSpA was compared. They suggested that ldCT could serve as a suitable method for screening axSpA patients, particularly in situations where access to MRI is limited or when clinical suspicion of the disease is high despite negative conventional radiographs. In childhood, the most significant challenge in using radiological tests during diagnosis or follow-up is potential radiation exposure. Advancements in CT technology have enabled the use of progressively lower radiation doses for various routine clinical indications without compromising diagnostic accuracy. For this purpose, ldCT stands out among the technologies. Chahal et al^10^ conducted a study to assess radiation exposure to the sacroiliac joint by comparing ldCT with radiography. They found that performing ldCT on the SI joint yields radiation doses of less than 1 mSv, a level comparable to that of direct radiography.^10^ Furthermore, CT offers better tight collimation than direct radiography, reducing scattered radiation and minimizing radiation exposure to the gonads.^10^ In the present study, all patients were examined with ldCT scanner as the effective dose to which the patient was exposed was 0.615 mSv.

While ldCT and radiography can identify structural damage, MRI of the SI joint has emerged as the most suitable imaging modality for diagnosing and classifying early inflammatory lesions. Our results confirmed that ldCT is superior to MRI in detecting structural alterations. Erosion was detected on MRI in only 11 of the 23 patients with erosion identified on ldCT. One of the primary treatment goals in childhood is to address the disease before the development of permanent structural lesions, aiming to transition the patient to adulthood with minimal sequelae. Therefore, it is important to detect early inflammatory changes before structural damage develops. MRI is particularly effective in detecting inflammatory changes in and around joints before any structural alterations occur.^9^ However, assessing density changes using HU may provide valuable data when evaluating bone marrow oedema. The HU functions as a relative quantitative measure of radiodensity, utilized by radiologists for analysing CT images.^6^ The utility of ldCT for the detection of bone marrow oedema in the SI joint was not tested before. However, previous adult studies have shown that the measurement of trabecular bone density through vertebral HU correlates with ldCT absorptiometry.^11^^,^^12^ Marques et al^11^ focused on assessing the correlation between HU density on ldCT and oedema on MRI. They demonstrated that ldCT revealed a decreased density in the vertebrae where the lesion was observed on MRI. Correspondingly, in our study, we assessed a structure to the sacroiliac joint, which is amorphous and lacks more than one equivalent level. Herein, two radiologists measured HU within regions of subchondral oedema identified on MRI by consensus. We observed lower density on ldCT in regions exhibiting heightened signal intensity on MRI, suggesting a potential correlation between density measurements on ldCT and signal intensity on MRI. We considered that ldCT could offer semi-quantitative data suitable for evaluating oedema, even though oedema cannot be directly determined on CT images in daily practice. We attributed the density loss to oedema and increased water density in that area. It is accurate to state that we utilized MRI as a reference for identifying potential areas of oedema in ldCT. However, based on the findings of our study, the current data is not sufficient to recommend that ldCT can replace MRI in this regard. It should be noted that research on imaging modalities is still in its preliminary stages, serving as foundational work towards the development of standardized procedures in these methods.

Our study's limitations include its single-centre design and the small sample size. In addition, the study was conducted by radiologists who are experts in the field of MSK and have not been tested for use by general radiologists who are not experts in this field. However, it is well known that in the evaluation of CT, the inter-reader correlation was higher, while MRI showed lower correlation in this regard.^13^

In conclusion, we suggest that the semi-quantitative density data acquired from ldCT can be employed to evaluate oedema on CT. However, it is crucial to note that this does not imply the direct visualization of oedema on CT. Instead, we demonstrate the feasibility of studying this aspect using ldCT, and we suppose that our study may provide preliminary data on the development of ldCT techniques. Furthermore, ldCT is superior to MRI in early structural change detection in paediatric case.

## Data Availability

The datasets used during the current study are available from the corresponding author on reasonable request.
